# Prognostic Value of *PLAGL1*-Specific CpG Site Methylation in Soft-Tissue Sarcomas

**DOI:** 10.1371/journal.pone.0080741

**Published:** 2013-11-15

**Authors:** Anne-Lise Peille, Veronique Brouste, Audrey Kauffmann, Pauline Lagarde, Valerie Le Morvan, Jean-Michel Coindre, Frederic Chibon, Laurence Bresson-Bepoldin

**Affiliations:** 1 Institut Bergonié, Comprehensive Cancer Centre, Bordeaux, France; 2 Univ. Bordeaux, Bordeaux, France; 3 INSERM U916 VINCO, Bordeaux, France; 4 Clinical and Epidemiological Research Unit, Institut Bergonié, Comprehensive Cancer Centre, Bordeaux, France; 5 Department of Bioinformatics, Institut Bergonié, Comprehensive Cancer Centre, Bordeaux, France; 6 Department of Biopathology, Institut Bergonié, Comprehensive Cancer Centre, Bordeaux, France; University of Navarra, Spain

## Abstract

Soft tissue sarcomas (STS) are rare, complex tumors with a poor prognosis. The identification of new prognostic biomarkers is needed to improve patient management. Our aim was to determine the methylation status of the 118 CpG sites in the *PLAGL1* tumor-suppressor gene P1 CpG island promoter and study the potential prognostic impact of *PLAGL1* promoter methylation CpG sites in STS. Training cohorts constituted of 28 undifferentiated sarcomas (US) and 35 leiomyosarcomas (LMS) were studied. *PLAGL1* mRNA expression was investigated by microarray analysis and validated by RT-qPCR. Pyrosequencing was used to analyze quantitative methylation of the *PLAGL1* promoter. Associations between global promoter or specific CpG site methylation and mRNA expression were evaluated using Pearson’s product moment correlation coefficient. Cox univariate and multivariate proportional hazard models were used to assess the predictive power of CpG site methylation status. Sixteen CpG sites associated with *PLAGL1* mRNA expression were identified in US and 6 in LMS. Statistical analyses revealed an association between CpG107 methylation status and both overall and metastasis-free survival in US, which was confirmed in a validation cohort of 37 US. The exhaustive study of P1 *PLAGL1* promoter methylation identified a specific CpG site methylation correlated with mRNA expression, which was predictive for both metastasis-free and overall survival and may constitute the first US-specific biomarker. Such a biomarker may be relevant for identifying patients likely to derive greater benefit from treatment.

## Introduction

 Sarcomas are uncommon tumors of mesenchymal origin, responsible for approximately 1-2% of adult cancers. They are currently classified according to their differentiation line. However, three main groups of STS have been described on a genetic level: a simple genetic profile, exclusively composed of well/dedifferentiated liposarcomas [1–3], a complex genomic profile observed in leiomyosarcomas (LMS) and undifferentiated sarcomas (US) [2,4,5], and a profile characterized by translocations [6]. These genetic alterations induce tumor development by targeting tumor suppressors (*p53*, *pRb*) [7,8] and genes related to ploidy status and proliferation. 

It has now been clearly established that changes in DNA methylation occur early in tumorigenesis. Cancer cells are characterized by global DNA hypomethylation, which promotes chromosomal breaks and rearrangements, and the silencing of tumor-suppressor genes, based on hypermethylation of their promoters [9][10]. 

*PLAGL1* is a maternally-imprinted gene, which maps to the human chromosome 6q24.2, a region often rearranged in many cancers [11–13], including sarcomas [14]. *PLAGL1* was first described as a tumor-suppressor gene as it showed anti-proliferative properties and shared the ability to concomitantly regulate apoptosis and cell-cycle arrest with p53 [15] [16,17]. However, recent studies have described an oncogenic role of *PLAGL1* in glioblastomas [18] and rhabdomyosarcomas [19], suggesting that *PLAGL1* functions depend on the cell context. Except in peripheral blood cells [20], *PLAGL1* expression is conditioned by methylation of a promoter (P1) containing a CpG island consisting of 118 CpG sites [21]. Loss of *PLAGL1* expression, due to either loss or hypermethylation of the active paternal allele, has been reported in various tumors [22]. 

The main treatment of STS combines primary radical surgery, chemotherapy, and radiation. Patient therapy depends mostly on the stage of the disease, however, the 5-year survival rate for STS patients is approximately 50%. Among STS subtypes, LMS and US exhibit the highest metastatic relapse rate and, consequently, the poorest prognosis. Therefore, it is necessary to develop new prognostic molecular biomarkers for future patient management. DNA methylation has been proposed as an alternate pathway to gene deletion and mutation-induced tumors. Surprisingly, in STS, while extensive studies have explored genetic alterations, the methylation status of specific genes involved in oncogenesis have yet to be elucidated [23–25]. Even if *PLAGL1* is frequently altered in various cancer types, to date, no study has extensively investigated the possible prognostic role of *PLAGL1* promoter methylation status in STS and its possible association with cancer patient outcome.

This work investigated the putative clinical prognostic role of specific CpG-site methylation levels of the *PLAGL1* promoter in leiomyosarcomas and undifferentiated sarcomas. A strategy was developed for performing an exhaustive study of the methylation levels of the 118 CpG sites on the *PLAGL1* P1 CpG island using pyrosequencing. This approach determined the methylation profile of each CpG site of the P1 CpG island and identified specific CpG sites particularly correlated with *PLAGL1* mRNA expression and clinical outcomes. 

## Materials and Methods

### 1. Patients and ethics statement

The initial study cohort of 63 sarcoma samples included 35 LMS and 28 US, and a second cohort of 37 US was used to validate the prognostic value of CpG sites. All samples were primary tumors from patients who had not been previously treated with radiotherapy or chemotherapy and exhibited no other diagnosed cancers. All tumors were provided by the French Sarcoma Group (GSF) database, part of the Conticabase (www.conticabase.org), containing data from adult soft tissue sarcomas [26]. All samples studied were fresh-frozen tumors which exhibited a high tumor-cell content (>80%). All cases were histologically reviewed by the pathologist subgroup and classified according to the 2002 WHO classification. 

The samples used in this study formed part of the Biological Resources Center of the Institut Bergonié Comprehensive Cancer Center (CRB-IB). In accordance with the French Public Health Code (articles L. 1243-4 and R. 1243-61), the CRB-IB has been approved by the French authorities to deliver samples for scientific research (number AC-2008-812, on February 2011). These patient samples were requalified for research. The patients signed an informed consent form approved by the Committee for the Protection of Individuals (CPP).

### 2. *PLAGL1* mRNA expression analysis

 The expression of *PLAGL1* mRNA in sarcoma samples was studied using data obtained with the Human Genome U133 plus 2.0 array (Affimetrix) by Chibon et al. [26] and deposited at Gene Expression Omnibus: accession number GSE21050. *PLAGL1* mRNA expression was assessed by three probe sets which were found to be highly correlated (> 0.97 Pearson's Correlation Coefficient, Figure S1). The mean of the GCRMA normalized expression values of the three probes was calculated and used to identify associations with the methylation of CpG sites and clinical factors.

*PLAGL1* mRNA expression data were then confirmed by RT-qPCR in tumors where total RNA was available. For RT-qPCR, 400 ng total RNA were reverse transcribed in a 20 µl mixture to generate first-strand cDNA using the high-capacity cDNA reverse-transcription site kit (Applied Biosystems) according to the manufacturer’s instructions. Reverse transcriptions were performed in triplicate. All qPCR were performed in a 20 µl final volume containing 20 ng cDNA preparation using power Sybr-green PCR master mix (Applied Biosystems) according to the manufacturer’s instructions and the Step-One plus real-time PCR system (Applied Biosystems). Primers for *PLAGL1*, *BACTIN*, and *PRPL0* were designed using Primer 3 software (Table S1). *BACTIN* and *PRPL0* were used for data normalization.

Correlations between mRNA expression values from microarray and RT-qPCR data were evaluated with GraphPad Prism v4 software, using Spearman’s product-moment correlation coefficient.

### 3. CGH array

Genomic profiling was performed using a DNA microarray developed in our laboratory. Three thousand eight hundred seventy-four BAC/PAC DNAs (BACPAC Resources Center, Children’s Hospital, Oakland Research Institute) were spotted in triplicate on ultraGAPS slides (Corning). The probes were prepared and hybridized as previously described [27]. The data were analyzed with software developed at Institut Curie (CAPweb, http://bioinfo-out.curie.fr/CAPweb/). Cyanine-5/cyanine-3 ratios between 0.8 and 1.2 were considered as normal status and ratios >1.2 and <0.8 were considered as gains and losses, respectively. Analysis of array-CGH (computation of genomic alterations) was provided by the VAMP interface (http://bioinfo.curie.fr/vamp) [28].

### 4. Promoter methylation analysis by pyrosequencing

The CpG island of the P1 *PLAGL1* promoter was defined by UCSC Genome Browser as follows: P1, 931 bp (144371540-144370610, 5’-3’) containing 118 CpGs (Figure S2). Quantitative methylation of all 118 CpGs was evaluated by pyrosequencing. 

Genomic DNA was isolated using a standard phenol-chloroform extraction protocol. DNA samples (100-500 ng), including positive controls for methylated and unmethylated status (EpiTect PCR *control DNA*; *Qiagen*, Hilden, Germany), were modified by sodium bisulfite treatment, using the Epitect bisulfite Kit (Qiagen, Hilden, Germany), according to the manufacturer's instructions. Bisulfite-modified DNA was then used as a template for PCR amplification.

PCR was carried out as follows: 1X PCR buffer, 200 μM of each dNTP, 1.5 to 2.5 mM MgCl_2_, 10-50 ng DNA, 200 nM of each primer, and 2 units Platinium Taq Polymerase (Invitrogen, CA). Initial denaturing for 3 min at 94°C was followed by 50 cycles, starting with a step at 94°C for 20 s. Different annealing temperatures were used according to the primers for 20 s (Table S1), followed by a 30-45 s step at 72° C, with a final extension at 72° C for 7 min. Primers used for PCR and sequencing are listed in Table S1.

Amplification and sequencing primers were designed for the bisulfite-converted DNA using PyroMark Assay Design Software 2.0 (Qiagen) and synthesized by Eurogentec (France). To account for DNA fragmentation introduced by bisulfite treatment, 4 different amplification reactions (A0 to A3) were designed to cover all 118 CpG sites. Since the optimum pyrosequencing reading length is 60-120 nucleotides per primer, more than one sequencing primer was needed to cover the multiple CpG sites contained in the various amplicons (Figure S2, Table S1). Pyrosequencing was performed using the PyroGold kit and PSQ 96 ID instrument (Qiagen), as directed by the manufacturer. CpG sites were quantified using Pyro Q-CpG 2.0 methylation software (Qiagen).

The coefficient of variance for methylation levels of CpG sites was calculated using demethylated and hypermethylated control DNA supplied by the manufacturer. As expected, it was < 2%. 

### 5. Statistical analysis

Associations between global promoter or specific CpG site methylation and mRNA expression were evaluated with R software, version 2.13.0, using Pearson’s product-moment correlation coefficient. 

A screening analysis was performed using moderated t-tests with the p-value adjusted according to the Benjamini and Hochberg method for False Discovery Rate (FDR) and the R/Bioconductor package limma [29] to identify CpG sites associated with clinical factors in the 28 US and 35 LMS from the tumor training sets. The association of CpG-site methylation with clinical prognosis, such as overall (OS) and metastasis-free survival (MFS) was confirmed using Cox univariate analysis. Duration of OS was calculated from the date of diagnosis until the date of last follow-up or death from any cause. MFS was calculated from the date of first diagnosis until the date of first metastasis. Patients who were alive or dead without metastasis at the last follow-up were censored. All variables significant at p<0.05 in the univariate analyses were included in stepwise, ascending, Cox-regression models. To validate the prognostic value of previously-identified CpG sites in terms of OS or MFS, Cox univariate analyses were performed on a second cohort (validation set) of 37 US with a pre-fixed cut-off, defined as the median methylation percentage of the first cohort. SPSS software version 16.0 was used for statistical analyses. Survival curves were computed by the Kaplan-Meier method. *P* ≤ 0.05 was considered significant. 

## Results

### 1. Clinical characteristics of patients

Primary STS tumors were obtained before any treatment. Thirty-five LMS and 28 US, constituting the training sets, were randomly selected from 52 LMS and 65 US included in the Chibon et al., study [26]. The remaining 37 US were studied as a validation set. The clinical characteristics of the samples are described in Table 1. In LMS, median OS was 37 months (range: 4-296 months) and median MFS was 15 months (0-92 months). Tumor grade, size, and differentiation stage were not associated with MFS or OS of LMS. In US, median OS was 40 months (range: 2-203 months) and median MFS was 13 months (from 0 to 52 months). Median OS in the US validation set was 28 months (4-166 months) and median MFS was 17 months (range: 0-166 months). In both cohorts of US tumors, tumor grade and size were not associated with MFS or OS (Tables 2 and 3). In the three cohorts studied, patient age was significantly associated with overall survival (in LMS: *P*=0.025, HR=2.75; in US-training set: *P*=0.021, HR=4.3; in US-validation set: *P*=0.034, HR=3.9) (Tables 2 and 3). The differentiation stage could not be tested due to small number of patients with low differentiation scores. 

**Table 1:** Patient Demographics and Clinical Characteristics.

		Leiomyosarcomas	Undifferentiated sarcomas	
		Training set(n =35)	Training set (n =28)	Validation set (n=37)
Gender				
	male	19	17	24
	female	16	11	13
Year of diagnosis		1989-2005	1992-2006	1985-2007
Median age at diagnosis ±SDⱡ		60.5 ± 17 ^$^	62.5 ± 15 ^$^	64 ± 15 ^$^
Median tumor size (mm)		100	85	80
Tumor size range (mm)		40-150	20-200	20-200
Complete remission after treatment		30	24	32
Relapse events				
	Local recurrences	7	5	10
	Metastasis	18	11	18
Differentiation score				
	G1 and G2	13	2	6
	G3	16	22	29
	ND	6	4	2
Primary tumors grade				
	G1 and G2*	14	7	7
	G3	20	20	29
	ND	1	1	1
CINSARC				
	C1	13	12	7
	C2	22	16	29
	ND	0	0	0
Median of mitotic index±SD		20±12	24±16	25±18

* Differentiation grades G1 and G2 are considered together as the cohort included too few G1 tumors.

^ⱡ^ SD standard deviation. ND not determined

^$^ significantly associated with overall survival, p<0.05; (n=): number

**Table 2:** Univariate and multivariate Cox analyses for metastasis-free survival in US.

***metastasis free survival***													
			Training set								Validation set		
			univariate analysis				multivariate analysis				univariate analysis		
			*P* value	HR	IC 95%		*P* value	HR	IC 95%		*P* value	HR	IC 95%
Grade 3			0.81	1.18	[0.34-4.44]						0.74	0.83	[0.27-2.53]
Tumor size			0.1	2.8	[0.81-9.66]						0.92	0.95	[0.36-2.51]
Median *PLAGL1* expression value			***0.035***	4.23	[1.11-16.17]		NR				0.73 0.39	1.06 1.5	[0.80-1.37] 0.59-3.79
CINSARC			***0.025***	5.817	[1.24-27.26]		NR				ND		
Mean methylation percentage of all CpGs			0.887	1.1	[0.27-4.45]								
CpG8 (≤ 44.06)			***0.041***	0.202	[0.04-0.94]		NR				0.89	0.93	[0.305-1.042]
CpG 46 (≤ 22.7)			***0.026***	0.169	[0.03-0.81]		NR				ND		
CpG 49 (≤ 23.99)			***0.03***	0.251	[0.07-0.87]		***0.027***	5.85	[1.224-27.957]		ND		
CpG 61 (≤ 25.6)			0.313	0.531	[0.15-1.82]								
CpG 78 (≤ 26.9)			0.158	0.41	[0.12-1.41]								
CpG 80 (≤ 26.39)			0.548	0.686	[0.2-2.35]								
CpG 81 (≤ 28.65)			0.533	0.676	[0.19-2.32]								
CpG 87 (≤ 33.41)			0.117	0.345	[0.09-1.31]								
CpG 91 (≤ 35.61)			0.117	0.345	[0.09-1.32]								
CpG 92 (≤ 33.78)			0.095	0.319	[0.08-1.22]								
CpG 96 (≤ 32.08)			0.052	0.218	[0.05-1.01]								
CpG 97 (≤ 27.52)			0.254	0.461	[0.12-1.74]								
CpG 106 (≤ 33.91)			0.075	0.298	[0.08-1.13]								
CpG 107 * (≤ 35)			***0.021***	0.16	[0.034-0.75]		***0.031***	5.601	[1.174-26.725]		***0.001***	6.526	[2.113-20.156]
CpG 108 (≤ 34.97)			0.123	0.351	[0.09-1.38]								
CpG 110 (≤ 30.54)			0.097	0.323	[0.08-1.23]								
CpG 113 (≤ 43.31)			0.234	0.472	[0.14-1.63]								
CpG 114 (≤ 38.08)			0.131	0.359	[0.09-1.36]								

* Associated with *PLAGL1* mRNA expression. p<0.05 was considered significant (values in bold and italics). Values in brackets=median of methylation percentage. ND: Not determined due to the imbalance between groups. NR: not retained by stepwise ascending Cox regression models.

**Table 3:** Univariate and multivariate Cox analyses for overall survival in US.

***overall survival***												
		Training set								Validation set		
		univariate analysis				multivariate analysis				univariate analysis		
		*P* value	HR	IC 95%		*P* value	HR	IC 95%		*P* value	HR	IC 95%
Grade 3		0.87	1.11	[0.34-3.63]						0.67	0.76	[0.21-2.7]
Tumor size		0.37	1.6	[0.56-4.7]						0.56	1.35	[0.48-3.75]
Median *PLAGL1* expression value		***0.049***	3.16	[1.003-9.98]		NR				0.69	1.06	[0.79-1.40]
CINSARC		***0.047***	3.784	[1.02-14.08]		NR				ND		
age		***0.021***	4.3	[1.24-14.7]		***0.006***	6.686	[1.72-25.94]		***0.034***	3.95	[1.11-14.1]
Mean methylation percentage of all CpGs		0.65559	1.36	[0.33-5.73]								
CpG 17 (≤ 23.71)		0.6	1.343	[0.45-4.04]								
CpG 106 (≤ 33.91)		0.067	0.336	[0.10-1.08]								
CpG 107* (≤ 35)		***0.032***	0.27	[0.09-0.89]		NR				***0.001***	9.26	[2.5-33.8]
CpG 110 (≤ 30.54)		***0.045***	0.299	[0.09-0.97]		***0.012***	5.057	[1.42-17.95]		0.716	0.751	[0.16-3.5]
CpG 113 (≤ 43.31)		0.054	0.319	[0.01-1.02]								
CpG 114 (≤ 38.08)		0.375	0.601	[0.19-1.85]								

* Associated with *PLAGL1* mRNA expression. p<0.05 was considered significant (values in bold and italics). Values in brackets=median of methylation percentage. ND: Not determined due to the imbalance between groups. NR: not retained by stepwise ascending Cox regression models.

Tumors were also classified according to the CINSARC (Complexity INdex in SARComas) scoring system [26] where C1, the lower CINSARC score, indicates a good prognosis and C2, the higher CINSARC score, corresponds to a poor prognosis. In LMS, CINSARC was associated with MFS (*P*=0.003, HR=9.8). In the US training set, CINSARC was associated with MFS (*P*=0.025, HR=5.8) and OS (*P*=0.047, HR=3.8). In the US validation set, CINSARC appeared to be associated with OS, although the values were not statistically significant (*P*=0.12, HR=5), but it was impossible to confirm the association with MFS due to the absence of metastasis in the C1 group (Tables 2 and 3).

### 2. *PLAGL1* expression in STS

*PLAGL1* mRNA expression was analyzed in 35 leiomyosarcomas (LMS) and 28 undifferentiated sarcomas (US), using the data obtained by Chibon et al [26] with the Human Genome U133 plus 2.0 array (Affimetrix). Prior analysis, we confirmed array-based *PLAGL1* mRNA expression measures by RT-qPCR. Good correlation was observed between microarray expression data and RT-qPCR (Spearman r=0.93, p<0.0001 normalized by *BACTIN* and r=0.86, p<0.0001 normalized by *RPLP0*) (Figure S3), thus validating the microarray *PLAGL1* mRNA expression values. 

**Figure 1:**
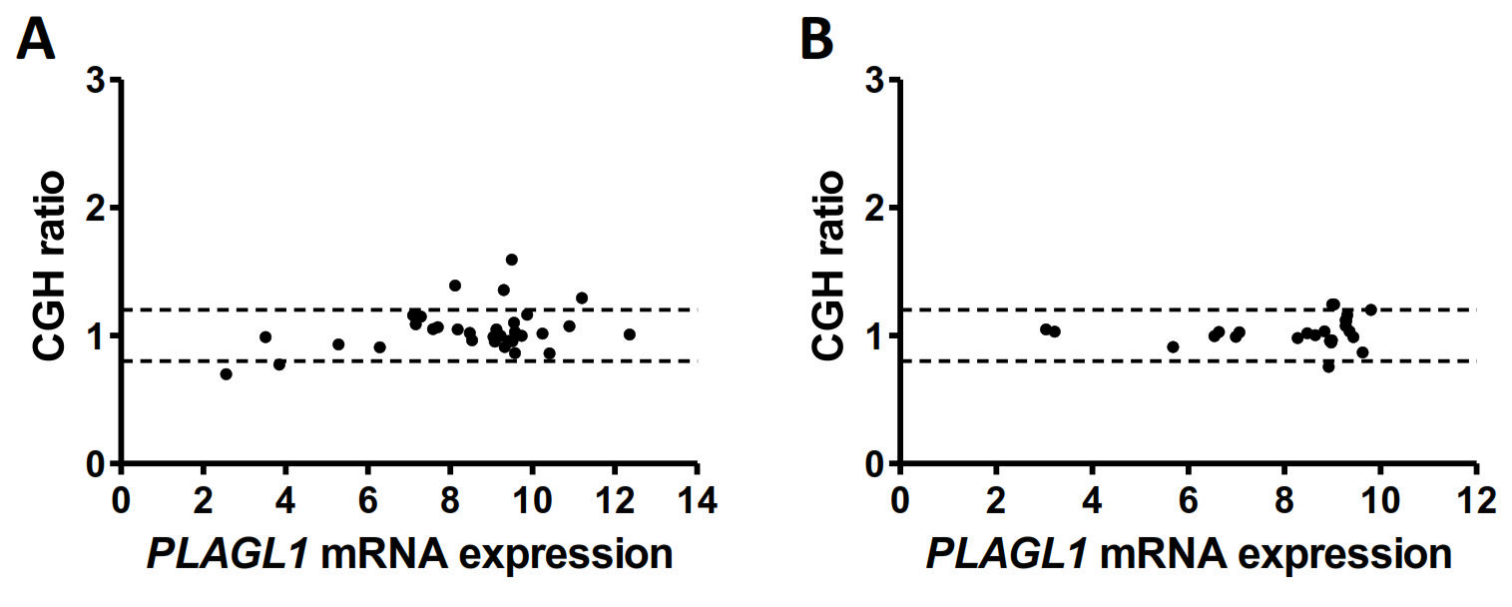
Correlation between *PLAGL1* genomic status assessed by CGHarray and *PLAGL1* mRNA expression assessed by Affymetrix HGU 133 plus 2.0 array in LMS (A) and US (B). *PLAGL1* mRNA expression was evaluated by microarray (mean of the 3 Affimetrix probes) and plotted in log_2_. CGH ratios between 0.8 and 1.2 (dotted lines) were considered as normal status, and ratios >1.2 and <0.8 were considered as gains and losses, respectively.

*PLAGL1* mRNA expression was not significantly different in LMS and US in terms of the median (9.07 in LMS and 8.96 in US) or heterogeneity of the expression values, which ranged from 2.6 to 12.3 in LMS and from 3 to 9.8 in US. The well-known high level of chromosome alterations in sarcomas like LMS and US may explain this heterogeneity. To verify this hypothesis, we studied the correlation between *PLAGL1* mRNA expression and its genomic status, obtained by CGHarray. No significant alteration in the genomic profile of the locus containing the *PLAGL1* gene (6q24.2) was detected in US and, consequently, no correlation between the genomic profile and mRNA expression of *PLAGL1* was observed in these sarcomas (Pearson=0.131 *P*=0.533). In contrast, 6 out of 35 LMS exhibited chromosome alterations: *PLAGL1* was deleted in 2 tumors (ratio<0.8) and gained in 4 tumors (ratio>1.2) (Figure 1). These alterations correlated with *PLAGL1* mRNA expression, since *PLAGL1* expression increased in tumors exhibiting gene gain and decreased in those with gene deletion (Pearson= 0.354 *P*=0.043). Nevertheless, this phenomenon alone did not account for the heterogeneity of *PLAGL1* mRNA expression observed in all LMS, since a majority of the tumors had no gene alteration but exhibited a large range of *PLAGL1* mRNA expression (range 3.5 to 12.3). 

Thus, we investigated whether *PLAGL1* expression in STS was regulated by methylation of its promoter, which would account for its heterogeneity.

### 3. Promoter methylation and *PLAGL1* mRNA expression

**Figure 2:**
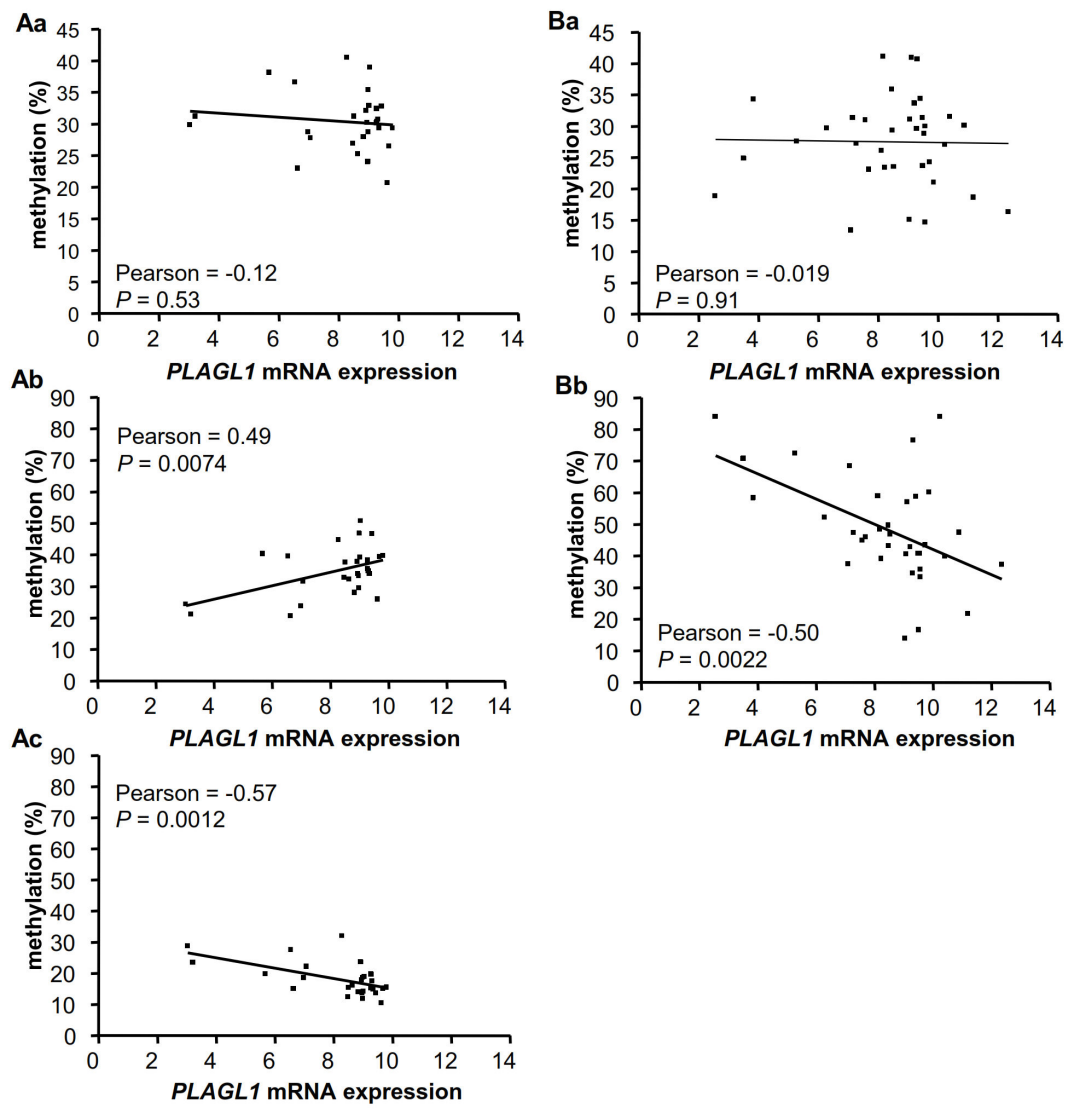
Pearson’s correlation between *PLAGL1* mRNA expression and the methylation percentage of CpGs in US and LMS. Correlation between *PLAGL1* mRNA expression and the mean methylation percentage of all CpG sites in undifferentiated sarcomas (US) (**Aa**) and leiomyosarcomas (LMS) (**Ba**). **Ab**: Correlation between *PLAGL1* mRNA expression and the mean methylation percentage of CpGs exhibiting positive correlation with *PLAGL1* mRNA expression in US. **Ac**: Correlation between *PLAGL1* mRNA expression and the mean methylation percentage of CpGs exhibiting negative correlation with *PLAGL1* mRNA expression in US; **Bb**: Correlation between *PLAGL1* mRNA expression and the mean methylation percentage of CpGs exhibiting negative correlation with *PLAGL1* mRNA expression in LMS. *PLAGL1* mRNA expression was evaluated by microarray (mean of the 3 Affimetrix probes) and plotted in log2. *P* ≤ 0.05 are considered significant.

Pyrosequencing was used to study the methylation status of all 118 CpG sites on the *PLAGL1* P1 CpG island promoter. We found that the mean methylation percentage of the *PLAGL1* P1 CpG island (mean methylation percentage of all CpG sites) was not significantly different in LMS and US in terms of the median methylation value (27.5±7% in LMS and 30.4±4.6% in US). However, a pronounced variation was observed in mean methylation values in the tumors, ranging from 13.4% to 41.1% in LMS and 20.7% to 40.7% in US. Thus, the next stage was to assess the correlation between the mean methylation percentage of the P1 CpG island and *PLAGL1* mRNA expression in US and LMS. No significant correlations were found in either tumor type (Pearson=-0.12, *P*=0.53 for US; Pearson=-0.019, *P*=0.91 for LMS) (Figure 2 Aa, Ba). Analysis of the percentage methylation of each CpG site on the P1 CpG island revealed highly variable values between two consecutive CpG sites in the same tumor and between the same CpG site in different tumors (Figure S4). This suggested that methylation of specific CpG sites may be particularly correlated with *PLAGL1* mRNA expression, rather than global *PLAGL1* promoter methylation. 

 Statistical analysis revealed that the methylation status of 6 and 16 out of the 118 CpG sites correlated with *PLAGL1* mRNA expression in LMS and US, respectively (Figure 2) (Table 4). As expected, in LMS, these CpG sites were negatively correlated with mRNA expression (Pearson=-0.50, *P*=0.0022) (Figure 2Bb), but in US, 3 CpG sites exhibited a positive correlation between their methylation status and *PLAGL1* mRNA expression (Pearson=0.49, *P*=0.0074) (Figure 2Ab), while 13 others showed a negative correlation (Pearson=-0.57, *P*=0.0012) (Figure 2Ac). These results suggest that the methylation status of only a few of the 118 CpGs in the P1 CpG island is relevant for mRNA expression of *PLAGL1* regulation in STS and that these CpG sites are tumor-type-specific. 

**Table 4:** Correlation between specific CpG site methylation and *PLAGL1* mRNA expression in US and LMS training sets.

US	Methylation range (%)	Pearson. Correlation	*P* value		LMS	Methylation range (%)	Pearson. Correlation	*P* value
CpG n°54	20-61	-0.567	0.00165		CpG n°1	17-92	-0.476	0.00388
CpG n°29	0-12	-0.517	0.00486		CpG n°7	14-100	-0.47	0.00441
***CpG n°58***	***15-44***	***0.48***	0.00978		CpG n°4	14-90	-0.455	0.00606
CpG n°107	12-45	-0.461	0.0134		CpG n°6	16-98	-0.413	0.0136
CpG n°33	0-45	-0.426	0.0239		CpG n°33	0-100	-0.359	0.0317
CpG n°32	0-26	-0.422	0.0252		CpG n°5	0-78	-0.361	0.0331
***CpG n°51***	***13-42***	***0.408***	0.031					
CpG n°105	25-60	-0.403	0.0333					
CpG n°109	26-60	-0.402	0.0338					
***CpG n°45***	***29-72***	***0.398***	0.0358					
CpG n°28	7-43	-0.398	0.0358					
CpG n°26	5-31	-0.393	0.0383					
CpG n°27	1-12	-0.388	0.0416					
CpG n°34	0-45	-0.378	0.0474					
CpG n°30	3-19	-0.378	0.0476					

Pearson ≥ ± 0.3 and *P* ≤ 0.05 are considered significant. CpG sites where methylation correlated positively with *PLAGL1* mRNA in US are shown in bold and italics.

### 4. Prognostic value of *PLAGL1* promoter methylation and mRNA expression

**Figure 3:**
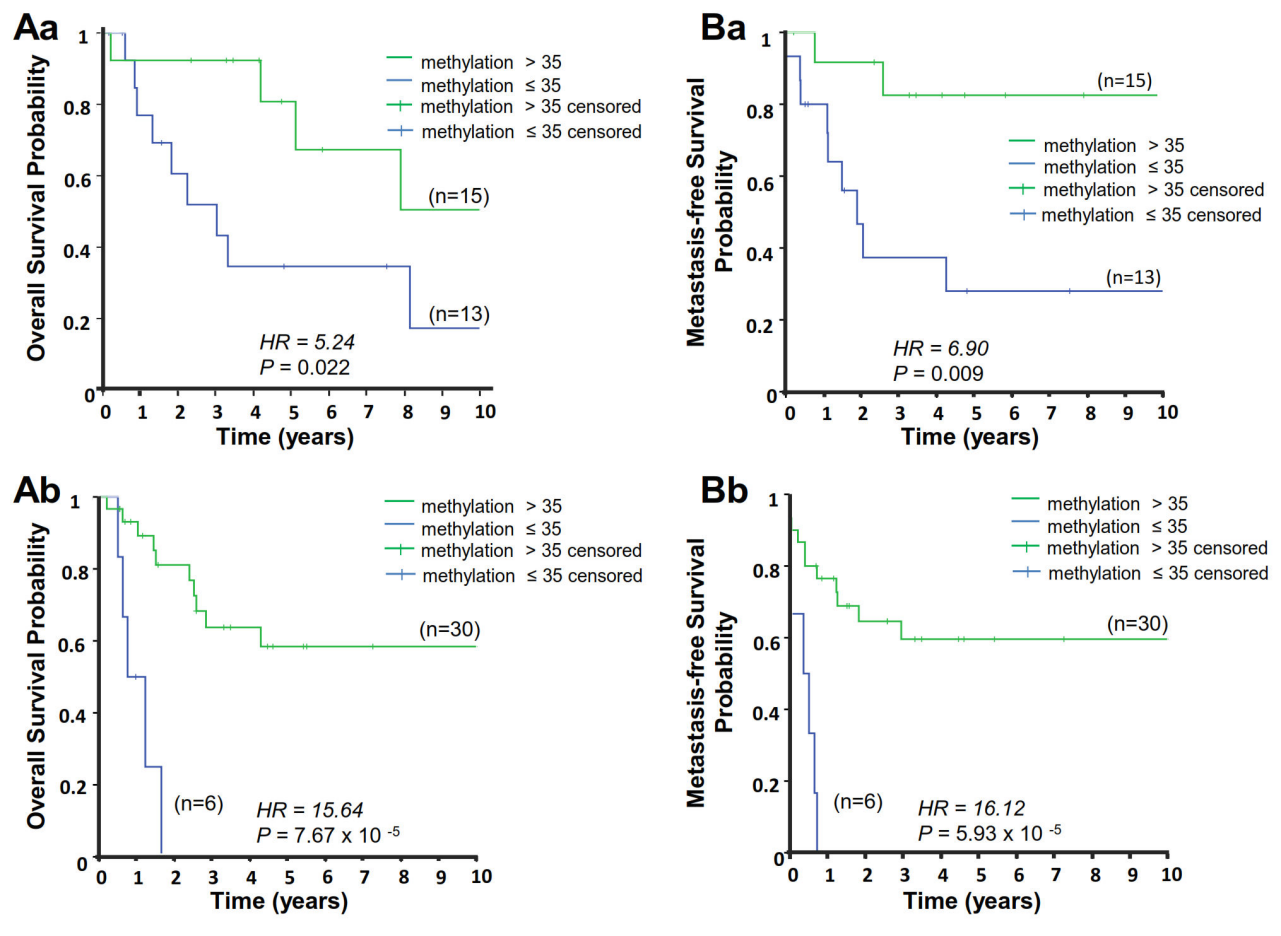
Prognostic value of CpG107 methylation status in US. Kaplan-Meier estimation of overall survival (**A**) and metastasis-free survival (**B**) according to CpG107 methylation status in the US training (**a**) and validation (**b**) sets. Median percentage methylation of CpG107 in the training set (35%) was defined as cut-off. This value was then applied to the validation set. *P* and HR values corresponded to the log-rank test comparing survival curves.*P* ≤ 0.05 are considered significant.

**Figure 4:**
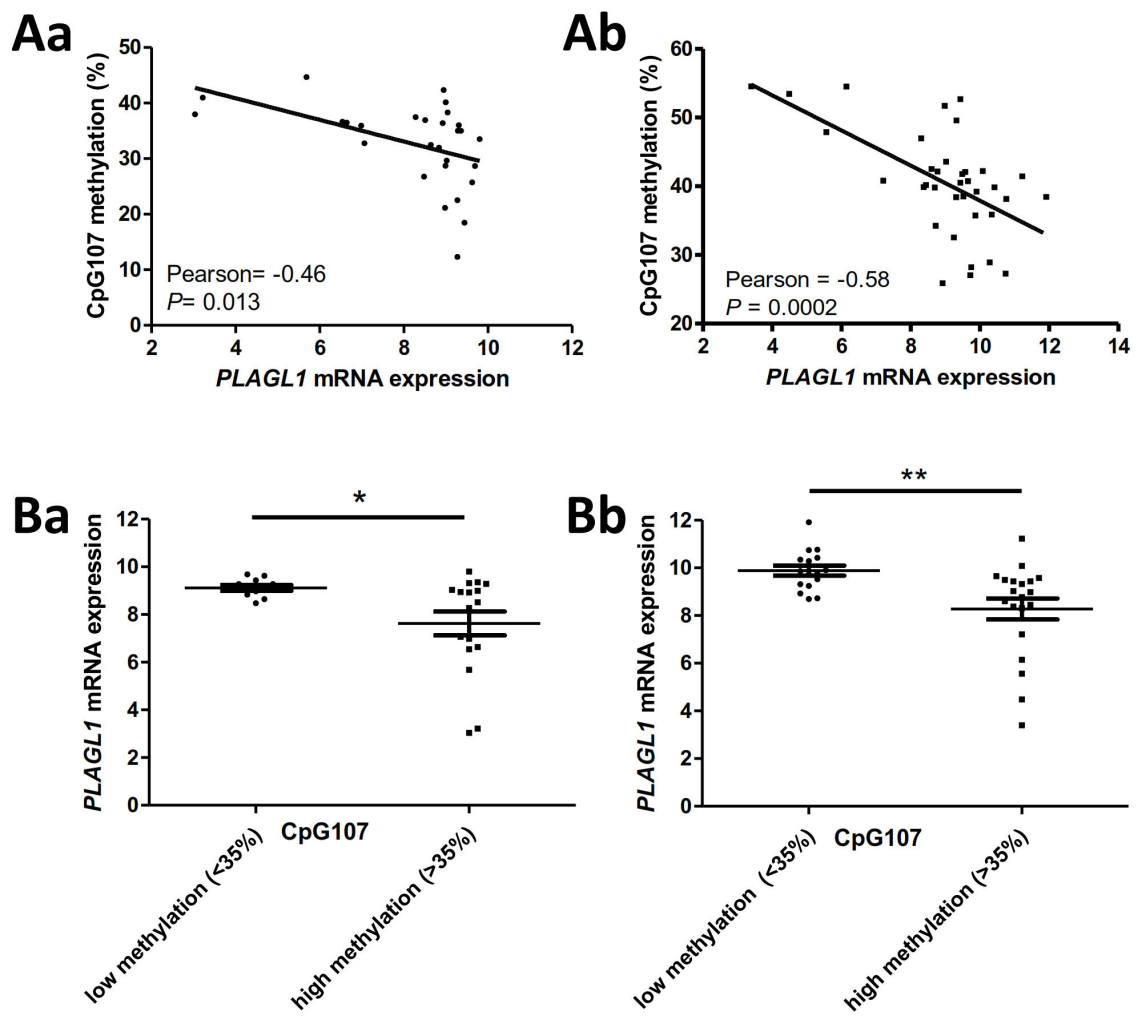
Relationship between CpG107 methylation percentage and *PLAGL1* mRNA expression. **A**: Pearson’s correlation between CpG107 methylation percentage and *PLAGL1* mRNA expression in tumors in the training (**a**) and validation (**b**) sets. *P* ≤ 0.05 are considered significant. **B**: *PLAGL1* mRNA expression of tumors in the training (**a**) and validation (**b**) sets according to CpG107 methylation status. Median percentage methylation of CpG107 in the training set (35%) was defined as cut-off. This value was then applied to the validation set. Statistical analyses were performed with the unpaired-t-test. *P<0.05, **P<0.01. *PLAGL1* mRNA expression was evaluated by microarray (mean of the 3 Affimetrix probes) and plotted in log_2_.

Investigation of a putative association of promoter methylation with STS clinical prognosis revealed that the mean methylation value of the *PLAGL1* promoter CpG island had no prognostic value in US (*P*=0.88, HR=1.1 for MFS; *P*=0.65, HR=1.36 for OS) (Tables 2 and 3) or LMS (*P*=0.77, HR=0.84 for MFS; *P*=0.14, HR=2.54 for OS). In contrast, screening analysis of associations between MFS or OS and the methylation status of each CpG site in US (see material and methods) revealed that CpG8, 46, 49, 61, 78, 80, 81, 87, 91, 92, 96, 97, 106, 107, 108, 110, 113, and 114 were associated with MFS (Table 2), while CpG17, 106, 107, 110, 113, and 114 were associated with OS (Table 3) (*P*≤0.05 and FDR≤30% were considered significant). Cox univariate analyses confirmed associations between MFS and CpG sites 8, 46, 49, and 107 (Table 2) and between OS and CpG 107 and 110 (Table 3). In LMS, the association between CpG19 and OS found during screening analysis was not confirmed with Cox univariate analysis (*P*=0.067), suggesting that *PLAGL1* P1 CpG site methylation is not prognostic in LMS. Methylation levels of significant CpG sites in a validation set of 35 US were studied to validate the association between CpG site methylation and clinical prognosis. The cut-off determined in the training set, corresponding to the median methylation level of each significant CpG site, was applied to the validation set. Associations between CpG107 and both MFS (*P*=0.001) (Table 2) and OS (*P*= 0.001) (Table 3) were confirmed in the validation set by univariate Cox analysis. The Kaplan-Meier method was used to produce survival curves stratified by DNA methylation (Figure 3). Low CpG107 methylation levels (<35%) were associated with poor prognosis for both MFS and OS in US. The methylation status of CpG107 showed significant concordance with mRNA expression in both cohorts (Pearson=-0.46, *P*=0.013 in training set, Pearson=-0.58, *P*=0.0002 in validation set) (Figure 4A). When analyzed by group, the low-methylation group consisted of a subset of samples with high mRNA expression, compared to the high-methylation group (unpaired t-test *P*=0.027 and *P*=0.0045 in training and validation set, respectively) (Figure 4B). Investigation of the prognostic value of *PLAGL1* mRNA expression in US revealed significant associations between *PLAGL1* mRNA expression and both overall and metastasis-free survival in the training set, but these results were not confirmed in the validation set (Tables 2 and 3). 

Given that more variables were significantly associated with OS and MFS in the training set than in the validation set, multivariate analyses were performed on the US training set to determine whether CpG107 was a significant independent prognosticator for MFS or OS. For MFS, CpG49 and CpG107 were significant markers in the multivariate analysis including CpG8, CpG46 *PLAGL1* mRNA expression and CINSARC (Table 2). For OS, CpG110 and age were significant markers in the multivariate analysis including CINSARC, *PLAGL1* mRNA expression, and CpG107 (Table 3).

## Discussion

LMS and US are rare, complex diseases and their pathogenesis is still poorly understood. They exhibit a high metastatic relapse rate leading to poor prognosis. The identification of new metastatic and survival prognosis markers is important for their management. The aims of this study were to evaluate the importance of individual *PLAGL1* promoter P1 CpG sites for *PLAGL1* mRNA expression and their potential association with sarcoma patient outcomes.

In this study, we identified CpG107 methylation status of the *PLAGL1* P1 promoter as the first prognostic biomarker for both MFS and OS in US. Methylation of the *PLAGL1* promoter was revealed to be heterogeneous along the CpG island and methylation of only a few CpG sites, differing according to STS subtypes, correlated with *PLAGL1* mRNA expression.

In US and LMS, transcriptomic data analysis revealed a high variability of *PLAGL1* expression, not entirely explained by amplification or deletion of the gene. This indicated that the methylation status of the P1 *PLAGL1* promoter in US and LMS may be responsible for the heterogeneity of *PLAGL1* expression in STS. The exhaustive study of the P1 *PLAGL1* promoter CpG island using the pyrosequencing method revealed the considerable heterogeneity of the *PLAGL1* methylation profiles of CpG sites along the CpG island and for a given CpG site in different tumors. Accordingly, we showed that the mean methylation percentage of all *PLAGL1* P1 CpGs was not correlated with mRNA expression levels, while methylation of only a few specific CpG sites was correlated, their number and localization varying according to the STS subtype studied. These data showed that most CpGs exhibit a large range of methylation compatible with mRNA expression values (Table 4). However, in US, some CpGs (CpG27, 29, 30, 32), located just before the transcription start site (TSS) (CpG32), exhibited a narrow range of methylation (0 to 20%). The specific promoter regions, called core regions, often situated around the TSS within a CpG island, have been shown to be crucial for regulating gene expression [30,31] and are probably more sensitive to small variations in methylation. Surprisingly, this study also revealed that 3 CpG sites (CpG45, 51, 58) were positively correlated with mRNA expression in US. This may be due to the fixation of transcriptional repressors since *in silico* studies using the TFsearch and PROMO databases identified CpG 45 and 51 as putative binding sites for P53, PAX5, and SP1, which may act as transcription repressor factors [32–34]. These results confirmed, as described in previous studies [35,36], that specific CpG sites were particularly relevant for directed mRNA expression. Importantly, these findings revealed that the CpG sites differed from one STS subtype to another, suggesting that the methylation modification of certain CpG sites may be a critical event for tissue-specific expression of *PLAGL1*. This may constitute a specific methylation signature, possibly resulting from a difference in tumor microenvironment [37] or differentiation lineage. These results demonstrate the relevance of performing methylation studies according to histological subtypes rather than in STS in general, even if large patient cohorts are difficult to constitute.

Epigenetic alterations are now clearly defined as early events during oncogenesis. Tumor-specific epigenetic alterations act as early, relatively stable molecular markers of malignancy, leading to better diagnosis, prognosis and therapy [10]. Investigation of the prognostic value of the methylation status of the *PLAGL1* promoter CpG island, showed that, as expected, the mean methylation value of the *PLAGL1* promoter had no prognostic value in either of these STS sub-types, possibly due to a lack of correlation between mean methylation value of the *PLAGL1* promoter and *PLAGL1* mRNA expression. In contrast, the screening analysis of the association between each CpG site's methylation value and clinical outcomes, followed by univariate Cox analyses, revealed that *PLAGL1* promoter methylation was a tumor type-specific biomarker. Indeed, while no CpG site was found to have any prognostic value in LMS, methylation of CpG107 was identified and validated as a prognostic factor for MFS and OS in US. Moreover, multivariate analysis showed that CpG107 methylation status was an independent prognosticator for MFS, suggesting that it is probably a reliable prognostic biomarker for US. Interestingly, CpG107 is one of the most strongly correlated with mRNA expression, suggesting that it is likely to be an important site for the transcriptional regulation of *PLAGL1* in US. The relevance of a specific, single, CpG site in mRNA expression and/or predictive of prognosis has recently been reported in various cancers. In liver cancer, the hypermethylation of one CpG silenced transcriptional expression of TTP [36] and, in chronic lymphatic leukemia, a single CpG dinucleotide has been identified to be important for ZAP-70 expression and prognosis [38]. Thus, these data highlight the interest of studying the detailed mapping of methylation patterns within a CpG island, as they revealed the complexity of gene regulation by the DNA methylation process and the relative importance of some CpG sites as clinical biomarkers. 

Low methylation (<35%) of CpG107 was correlated with significantly higher *PLAGL1* expression and poor prognosis. This is consistent with high *PLAGL1* mRNA expression having an adverse effect on survival in the training set. Together, these data suggest that the role of *PLAGL1* in US is more consistent with the phenotype of an oncogenic than a tumor-suppressor gene. Although *PLAGL1* was initially described as a tumor-suppressor gene, recent studies have shown its oncogenic role in glioblastomas [18] and rhabdomyosarcomas [19]. Moreover, the embryonic growth-retardation phenotype observed in *ZAC1*-deficient mice is unexpected for a putative tumor-suppressor gene [39] and more consistent with the phenotype of an oncogenic gene. Thus, *PLAGL1* functions apparently depend on the cell context and the biological role of *PLAGL1* in STS has yet to be established.

This study confirmed that the CpG-site methylation had a better prognostic value than mRNA expression since, in contrast to CpG site methylation, the prognostic value of mRNA expression was not confirmed in the validation set. Indeed, the clinical impact of biomarkers is dependent on the stability of the information provided. While DNA methylation is a covalent modification of the cytosine base and remains relatively stable *in vitro* and *in vivo* over time [40], RNA and protein expression results from a balance of distinct factors, including RNA polymerases, transcriptional activators and repressors, miRNA, and other processes leading to a significant variability in gene expression. Thus, recent studies have reported that DNA methylation analysis is a more accurate predictor of outcome than expression [38,41,42]. Finally, the possibility cannot be excluded that the methylation pattern of *PLAGL1* has an impact on the expression of other genes, for example by sequestering transcription factors. 

In conclusion, this research identified a specific CpG site where determination of the methylation status was associated with both metastasis-free and overall survival in undifferentiated sarcoma. Current guidelines recommend doxorubicin and iphosphamide-based chemotherapies for patients with STS, while their clinical outcomes remain quite diverse. In this context, the identification the methylation status of a single CpG may provide a useful epigenetic biomarker of primary US at increased risk of relapse and metastasis, thus improving patient management by selecting patients who would benefit from this treatment. Moreover, the methylation study of a single CpG site dramatically simplifies analysis, thus facilitating its application in pathology laboratories.

## Supporting Information

Figure S1 **Correlation between the expression values measured by the three PLAGL1 probe sets in the Human Genome U133 plus 2.0 array (Affimetrix).**
(TIF)

Figure S2 **Schematic diagram of *PLAGL1* gene and illustration of pyrosequencing assays.** **A**: Schematic diagram of *PLAGL1* gene in the 6q24.2 locus. The gray box represents the CpG island P1 of *PLAGL1* (931bp) and the black box represents the first non-coding exon. The CpG island of *PLAGL1* contains 118 CpG dinucleotides, represented by black vertical bars on the enlarged view of the CpG island (at the bottom of the figure). The asterisk identifies the transcription start site.
**B**: Illustration of pyrosequencing assays. CpGs contained in the CpG island P1 of *PLAGL1* are shown in bold underlined type. Each CpG is identified by a superscript number. PCR and sequencing primers are symbolized by full and dotted arrows, respectively. Four PCR were necessary to sequence the entire CpG island. The first assay required two sequencing primers (in blue, for dinucleotides CpG1 to 15). The second assay required three sequencing primers (in red, for dinucleotides CpG13 to 48). The third assay required four sequencing primers (in green, for dinucleotides CpG44 to 92). The fourth assay required four sequencing primers (in purple, for dinucleotides CpG77 to 118). **b**: biotinylated primer.
(TIF)

Figure S3 **Validation of *PLAGL1* mRNA expression by RT-qPCR.** Spearman’s correlation coefficient between *PLAGL1* mRNA expression evaluated by microarray (mean of three Affymetrix probes) and *PLAGL1* mRNA expression evaluated by RT-qPCR normalized by *BACTIN* (**A**) or *RPLP0* (**B**).
(TIF)

Figure S4 **Quantitative DNA methylation profiling of the CpG island of the P1 *PLAGL1* promoter analyzed by pyrosequencing.** Separate samples are organized in rows (LMS from 35 patients and US from 28 patients). Columns represent single CpG units. High methylation levels are depicted in red and low in dark blue.
(TIF)

Table S1 **Conditions of PCR reactions and list of primers used for qPCR, PCR and pyrosequencing reactions.**
(DOCX)
