# Supplementary figures and images for: Prognostic Value of PLAGL1-Specific CpG Site Methylation in Soft-Tissue Sarcomas
Source: PLoS One. 2013 Nov 15;8(11):e80741. doi: 10.1371/journal.pone.0080741 (PMC3829972; doi:10.1371/journal.pone.0080741)

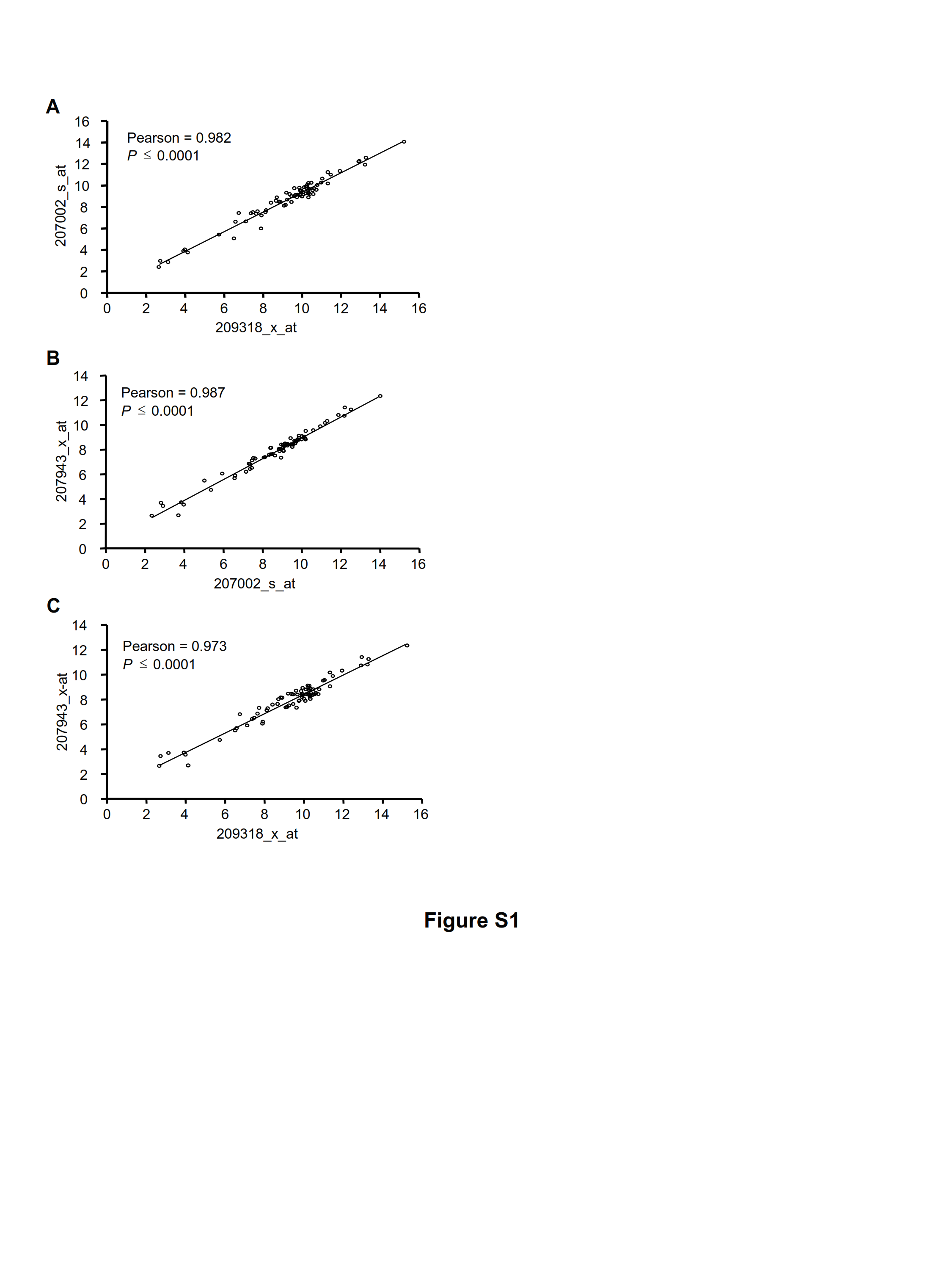

Supplement: Figure S1 — Correlation between the expression values measured by the three PLAGL1 probe sets in the Human Genome U133 plus 2.0 array (Affimetrix). (TIF) [file pone.0080741.s001.tif]

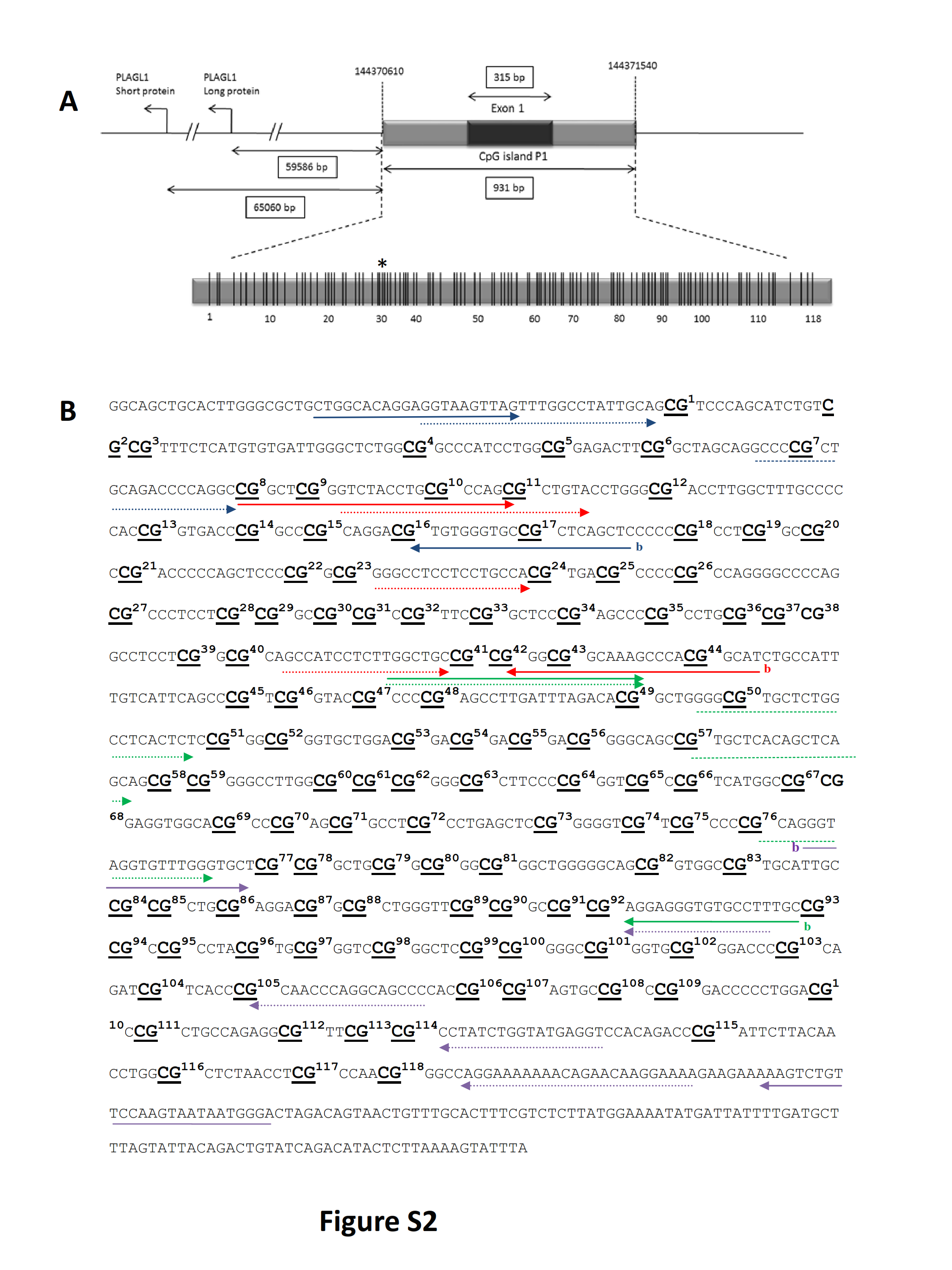

Supplement: Figure S2 — Schematic diagram of PLAGL1 gene and illustration of pyrosequencing assays. A: Schematic diagram of PLAGL1 gene in the 6q24.2 locus. The gray box represents the CpG island P1 of PLAGL1 (931bp) and the black box represents the first non-coding exon. The CpG island of PLAGL1 contains 118 CpG dinucleotides, represented by black vertical bars on the enlarged view of the CpG island (at the bottom of the figure). The asterisk identifies the transcription start site. B: Illustration of pyrosequencing assays. CpGs contained in the CpG island P1 of PLAGL1 are shown in bold underlined type. Each CpG is identified by a superscript number. PCR and sequencing primers are symbolized by full and dotted arrows, respectively. Four PCR were necessary to sequence the entire CpG island. The first assay required two sequencing primers (in blue, for dinucleotides CpG1 to 15). The second assay required three sequencing primers (in red, for dinucleotides CpG13 to 48). The third assay required four sequencing primers (in green, for dinucleotides CpG44 to 92). The fourth assay required four sequencing primers (in purple, for dinucleotides CpG77 to 118). b: biotinylated primer. (TIF) [file pone.0080741.s002.tif]

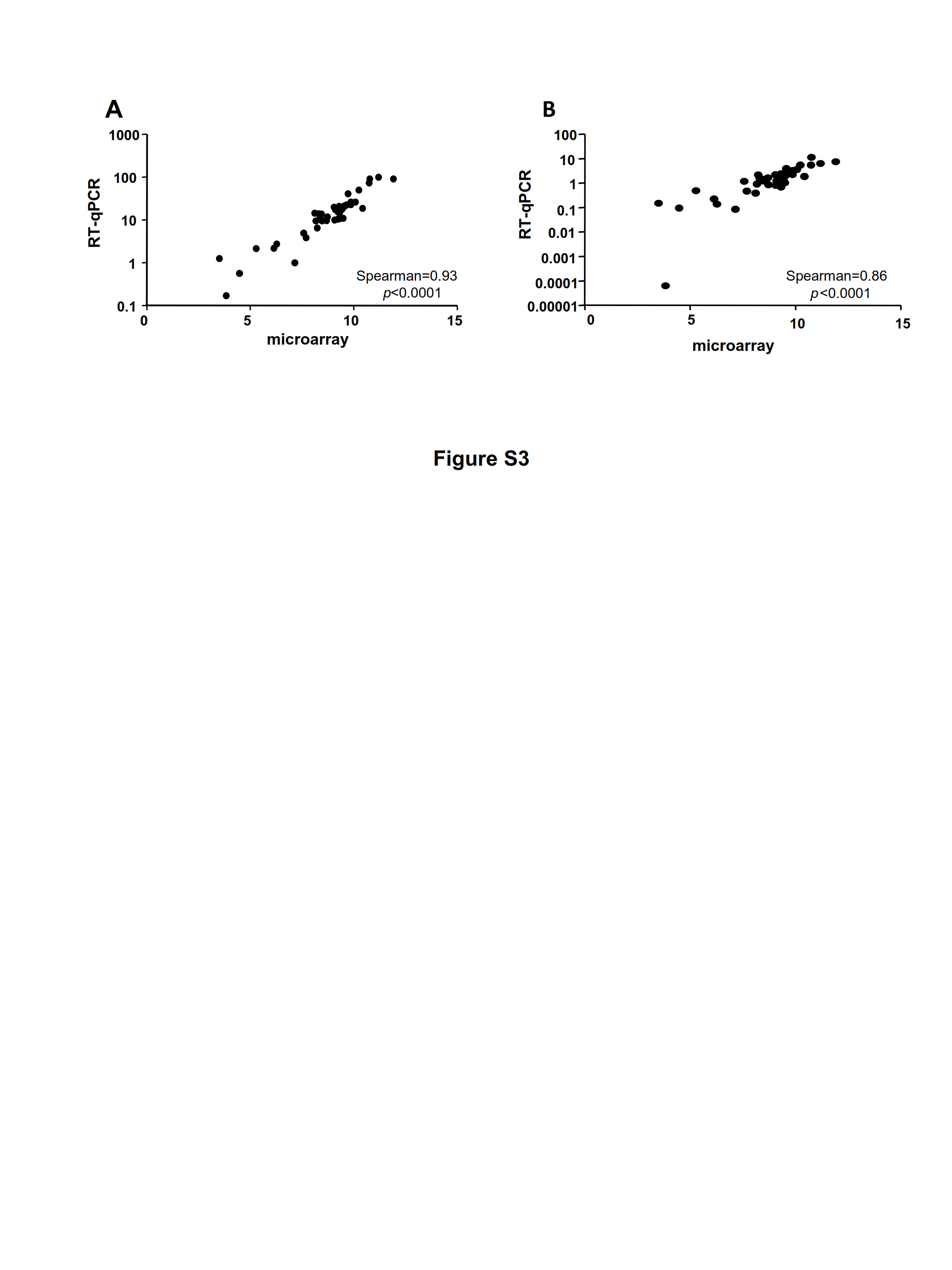

Supplement: Figure S3 — Validation of PLAGL1 mRNA expression by RT-qPCR. Spearman’s correlation coefficient between PLAGL1 mRNA expression evaluated by microarray (mean of three Affymetrix probes) and PLAGL1 mRNA expression evaluated by RT-qPCR normalized by BACTIN (A) or RPLP0 (B). (TIF) [file pone.0080741.s003.tif]

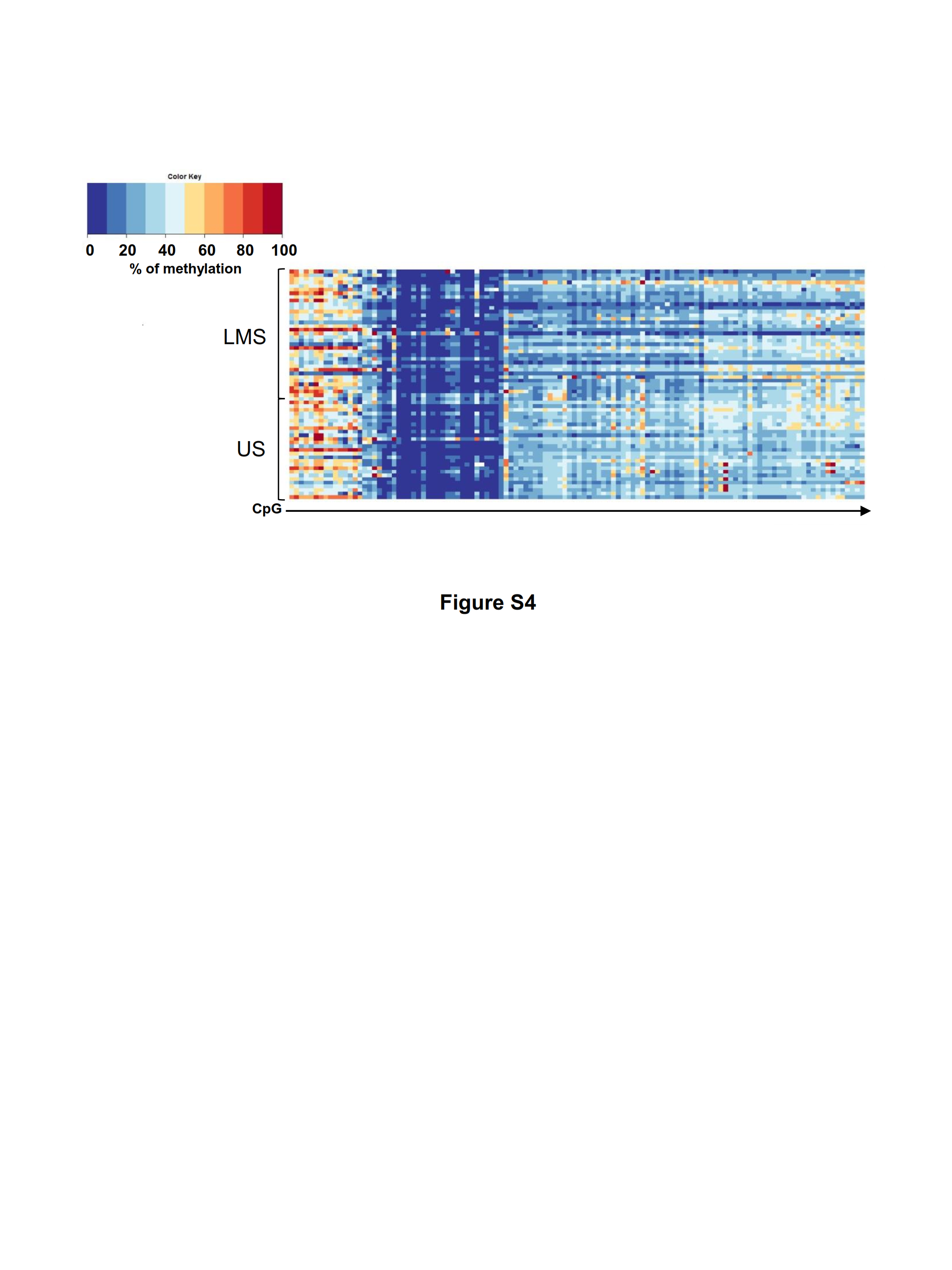

Supplement: Figure S4 — Quantitative DNA methylation profiling of the CpG island of the P1 PLAGL1 promoter analyzed by pyrosequencing. Separate samples are organized in rows (LMS from 35 patients and US from 28 patients). Columns represent single CpG units. High methylation levels are depicted in red and low in dark blue. (TIF) [file pone.0080741.s004.tif]
