# Supplementary material for: Prognostic Value of PLAGL1-Specific CpG Site Methylation in Soft-Tissue Sarcomas
Source: PLoS One. 2013 Nov 15;8(11):e80741. doi: 10.1371/journal.pone.0080741 (PMC3829972; doi:10.1371/journal.pone.0080741)
Supplement: Table S1 — Conditions of PCR reactions and list of primers used for qPCR, PCR and pyrosequencing reactions. (DOCX) [file pone.0080741.s005.docx]

| **primer name** | **sequence (5'-3')** | **annealing temperature and MgCl2 concentration** | **bases number** | **CpG sites** |
| --- | --- | --- | --- | --- |
| PLAGL1 A0 PCR FW | TTGTTGGTATAGGAGGTAAGTTAG | 54°C; 1.5mM | 24 |  |
| PLAGL1 A0 PCR RV biotinylated | AAACTAAACCACACCCACAC | 54°C; 1.5mM | 20 |  |
| PLAGL1 A0 sequencing 1 | GGTAAGTTAGTTTGGTTTATTGTA |  | 24 | 1-9 |
| PLAGL1 A0 sequencing 2 | GTTTTGTTGTAGATTTTAGGT |  | 21 | 8-15 |
| PLAGL1 A1 PCR FW | TGG TTT GGG TTT ATT TGT GTT AGT | 56°C; 1.5mM | 24 |  |
| PLAGL1 A1 PCR RV biotinylated | CCC CAA CCC TAT CTA AAT CAA AAC | 56°C; 1.5mM | 24 |  |
| PLAGL1 A1 sequencing 1 | GTT TAT TTG TGT TAG TGT TGT A |  | 22 | 13-25 |
| PLAGL1 A1 sequencing 2 | GGG TTT TTT TTT GTT A |  | 16 | 24-40 |
| PLAGL1 A1 sequencing 3 | GTA GTT ATT TTT TTG GTT GT |  | 20 | 41-48 |
| PLAGL1 A2 PCR FW | GGT TGT TGT GGG TGG TAA AG | 58°C; 2mM | 20 |  |
| PLAGL1 A2 PCR RV biotinylated | ACA AAA ACA CAC CCT CCT | 58°C; 2mM | 18 |  |
| PLAGL1A2 sequencing 1 | GGT TGT TGT GGG TGG TAA AG |  | 20 | 44-52 |
| PLAGL1 A2 sequencing 2 | GGG AGT GTT TTG GTT TTA TTT TT |  | 23 | 51-59 |
| PLAGL1 A2 sequencing 3 | GTG TTT ATA GTT TAG TAG |  | 18 | 58-78 |
| PLAGL1 A2 sequencing 4 | GTA GGG TAG GTG TTT GGG |  | 18 | 77-92 |
| PLAGL1 A3 PCR FW biotinylated | GGT AGG TGT TTG GGT GTT | 58°C; 2.5mM | 18 |  |
| PLAGL1 A3 PCR RV | CCC ATT ATT ACT TAA AAC AAA CTT | 58°C; 2.5mM | 24 |  |
| PLAGL1 A3 sequencing 1 | TTT CCT TAT TCT ATT TTT TTC CTA |  | 24 | 118-112 |
| PLAGL1 A3 sequencing 2 | CCT CAT ACC AAA TAA AC |  | 17 | 114-104 |
| PLAGL1 A3 sequencing 3 | AAA CTA CCT AAA TTA C |  | 16 | 105-91 |
| PLAGL1 A3 sequencing 4 | AAA CAC ACC CTC CTC |  | 15 | 92-77 |
| PLAGL1 qPCR FW | GCC TCA GTC ACC TCA AAA GC |  | 20 |  |
| PLAGL1 qPCR RV | CTT AAC CTG TGG GGC AAA GA |  | 20 |  |
| βactin qPCR FW | GGA CTT CGA GCA AGA GAT GG |  | 20 |  |
| βactin qPCR RV | AGC ACT GTG TTG GCG TAC AG |  | 20 |  |
| RPLP0 qPCR FW | TCG TGG AAG TGA CAT CGT CTT T |  | 22 |  |
| RPLP0 qPCR RV | CTG TCT TCC CTG GGC ATC A |  | 19 |  |

**Table S1: Conditions of PCR reactions and list of primers used for qPCR, PCR and pyrosequencing reactions.**
